# Psychiatric Comorbidity in Children with Autism Spectrum Disorders: A Comparison with Children with ADHD

**DOI:** 10.1007/s10826-012-9587-z

**Published:** 2012-05-01

**Authors:** Francisca J. A. van Steensel, Susan M. Bögels, Esther I. de Bruin

**Affiliations:** Research Institute of Child Development and Education, University of Amsterdam, Nieuwe Prinsengracht 130, 1018 VZ Amsterdam, The Netherlands

**Keywords:** ASD, ADHD, Children, Psychiatric comorbidity

## Abstract

The present study was conducted with the aim to identify comorbid psychiatric disorders in children with autism spectrum disorders (ASD) (*n* = 40) and to compare those comorbidity rates to those in children with attention deficit hyperactivity disorder (ADHD) (*n* = 40). Participants were clinically referred children aged 7–18 years. DSM-IV classifications were used for the primary diagnosis (ASD/ADHD), while comorbid psychiatric disorders were assessed using a structured diagnostic interview, the structured clinical interview for DSM-IV, childhood diagnoses (KID-SCID). Twenty-three children with ASD (57.5 %) had at least one comorbid disorder, whereas 16 children with ADHD (40.0 %) were classified as having at least one comorbid disorder. No group differences were found with respect to this comorbidity rate or for the rate of comorbid externalizing disorders (ODD and/or CD). However, children with ASD had more comorbid internalizing disorders compared to children with ADHD. More specifically, children with ASD had higher rates of anxiety disorders, but not mood disorders. No associations between comorbidity and age or between comorbidity and the intelligence quotient was found. It is important for clinicians to always be aware of, and screen for, comorbidity, and to consider treatment for these comorbid disorders. In addition, research should focus on establishing valid and reliable screening tools as well as effective treatment options for these comorbid disorders.

## Introduction

Children with autism spectrum disorders are characterized not only by their core deficits, impairments in social interaction and communication and repetitive stereotyped behaviors (APA, DSM-IV-TR 2000), but they also suffer from many comorbid features such as anxiety, depression, ADHD and behavioral problems (e.g. Leyfer et al. 2006). An important distinction, however, is the presence of comorbid symptoms versus comorbid disorders. For a full clinical picture of a child it is valuable to be informed with respect to his/her comorbid symptoms but often additional treatment options are more likely to be offered when a DSM-IV disorder is classified. While several studies have examined comorbid psychiatric symptoms in children with ASD (e.g. Gadow et al. 2005; Kim et al. 2000), studies that have examined the presence of comorbid psychiatric disorders are less common, particularly using standardized diagnostic interviews. We have identified ten of those studies (Table 1). In these studies, rates for at least one comorbid disorder vary between 63.3 % (Amr et al. 2012) and 96.4 % (Mukaddes et al. 2010). In addition, studies differ with respect to the most common disorder that is found, and rates for each specific disorder vary considerably (Table 1).

**Table 1:** Summary of studies that have examined the prevalence rates (%) of psychiatric comorbid disorders in children with ASD using (semi-) structured diagnostic interviews

First author	Year	Age range	ASD subtype and (sub)sample size	PSY	MDD	DYS	SOC	SAD	SPH	GAD	OCD	AGO	PAN	ADHD	ODD	CD
Amr	2012	4–11	AD (*n* = 55); PDD-NOS (*n* = 5)	63.3	13.3	NR	NR	8.3	**40.0** ^b^	10.0	**55.0** ^a^	NR	NR	**31.6** ^c^	NR	23.3
de Bruin	2007	6–12	PDD-NOS (*N* = 94)	80.9	10.6	2.1	11.7	8.5	**38.3** ^b^	5.3	6.4	6.4	1.1	**44.7** ^a^	**37.2** ^c^	9.6
Gjevik	2011	6–18	AD (*n* = 47); AS (*n* = 12); PDD-NOS (*n* = 12)	71.8	1.4	1.4	7.0	0.0	**30.9** ^a^	0.0	**9.8** ^c^	NR	NR	**30.9** ^a^	4.2	2.8
Leyfer	2006	5–17	AUT (*N* = 109)	73.0	10.1	NR	7.5	11.9	**44.3** ^a^	2.4	**37.2** ^b^	NR	0.0	**30.6** ^c^	7.0	NR
Mattila	2010	9–16	AS (*n* = 27); HFA (*n* = 23)	74.0	6.0	NR	4.0	2.0	**28.0** ^b^	NR	**22.0** ^c^	4.0	2.0	**38.0** ^a^	16.0	2.0
Mukaddes	2010	6–16	AS (*N* = 37)	96.7	**29.0** ^c^	NR	5.4	2.7	13.0	5.4	**32.0** ^b^	NR	5.4	**45.0** ^a^	5.4	5.4
Mukaddes	2010	6–20	AS (*n* = 30); HFA (*n* = 30)	94.0	21.7	1.7	13.3	13.3	**53.3** ^b^	10.0	**36.7** ^c^	NR	1.7	**65.0** ^a^	31.7	1.7
Simonoff	2008	10–14	AUT (*n* = 62); other ASD’s (*n* = 50)	70.8	0.9	0.5	**29.2** ^a^	0.5	8.5	13.4	8.2	7.9	10.1	**28.2** ^b^	**28.1** ^c^	3.2
Witwer	2010	6–17	AS (*n* = 16); AUT (*n* = 17); PDD-NOS (*n* = 26)	NR	14.8	13.1	16.4	14.8	**67.2** ^c^	24.6	4.9	NR	NR	**91.8** ^a^	**75.4** ^b^	49.2
Wozniak	1997	NR	AUT (*n* = 42); PDD-NOS (*n* = 24)	NR	**53.0** ^c^	9.0	9.0	17.0	19.0	20.0	16.0	28.0	4.0	**74.0** ^a^	**55.0** ^b^	13.0

The bold values were used to visually illustrate the most common (top-3) comorbid disorders

*AD* autistic disorder, *ADHD* attention deficit hyperactivity disorder, *AGO* agoraphobia, *AS* Asperger’s syndrome, *AUT* autism, *CD* conduct disorder, *DYS* dysthymic disorder, *GAD* generalized anxiety disorder, *HFA* high functioning autism, *MDD* major depressive disorder, *NR* not reported, *OCD* obsessive compulsive disorder, *ODD* oppositional defiant disorder, *PAN* panic disorder, *PDD-NOS* pervasive developmental disorder not otherwise specified, *PSY* at least one comorbid psychiatric disorder is present, *SAD* separation anxiety disorder, *SOC* social anxiety disorder, *SPH* specific phobia

^a^Most common comorbid psychiatric disorder

^b^Second most common comorbid psychiatric disorder

^c^Third most common comorbid psychiatric disorder

Factors that might be relevant to consider for their influence on comorbidity rates are gender, age and intelligence quotient (IQ). No significant gender differences in the rates of comorbid disorders were found in the study of Simonoff et al. (2008) and in the study of Gjevik et al. (2011). With respect to age, the study of Matilla et al. (2010) found that psychiatric comorbidity was more common in primary school-aged children with ASD compared to secondary school-aged children. Mixed results have been found for comorbidity and its relation with IQ: in the study of Simonoff et al. (2008) and the study of Gjevik et al. (2011), IQ was not associated with psychiatric comorbidity, while in the study of Witwer and Lecavalier (2010) children with an IQ below 70 had fewer psychiatric symptoms compared to children with an IQ above 70. Alternatively, children with ASD and an IQ below 70 may have poorer language skills to communicate (psychiatric) symptoms or internal feelings (to their caregivers), which could contribute to such findings.

Child psychiatric disorders can be roughly divided into two main groups: internalizing and externalizing (or disruptive behavior) disorders. Internalizing disorders are characterized by behaviors and emotions that are directed inwards and include mood disorders (e.g. major depressive disorder, dysthymic disorder) and anxiety disorders (e.g. separation anxiety disorder, social anxiety disorder, obsessive–compulsive disorder, specific phobia). Externalizing disorders are characterized by behaviors and emotions directed outwards and include ADHD, oppositional defiant disorder (ODD) and conduct disorder (CD). Since ADHD seems the most commonly reported comorbid disorder in children with ASD (Table 1), it is important to (1) explore differences and similarities between children with ASD and children with ADHD, and (2) to compare the prevalence of comorbid psychiatric disorders across those clinical groups. However, studies addressing these issues are rare. One study compared children with ADHD alone, children with ASD + ADHD and children with chronic multiple tic disorder + ADHD and found similarities as well as differences in comorbidity of symptoms among groups (Gadow et al. 2009). Similarities for all groups were found for ODD and CD symptoms, whereas differences between groups were found for anxiety symptoms. Children with ASD + ADHD were generally rated to have more severe anxiety symptoms compared to the other groups. In addition, Green and colleagues compared 20 adolescents with Aspergers’ Syndrome to 20 adolescents with CD and also found adolescents with Aspergers’ Syndrome to have more anxiety-related symptoms (Green et al. 2000).

To conclude, several studies have examined psychiatric comorbid disorders by using structured diagnostic interviews in children with ASD, however, comparative studies between ASD and other clinical groups (such as ADHD) on comorbid psychiatric disorders are rare. Examining comorbidity in clinical samples is important because they may influence the symptoms of the primary diagnosis (e.g. anxiety symptoms may exacerbate ASD-symptoms; Wood and Gadow 2010) and may affect treatment plans and outcomes (Matson and Nebel-Schwalm 2007). Furthermore, knowing which comorbid disorders are (more) likely to exist in a particular clinical sample, may lead to (early) screening activities and (the development of) prevention programs. Therefore, the current study aims to examine comorbid disorders in children with ASD and to compare them to a group of children with ADHD. In addition, the relation between comorbidity and age, and between comorbidity and IQ, was explored (gender was not examined because of power issues).

## Method

### Participants

All children were referred to a general outpatient mental health center in Maastricht, the Netherlands. In total 80 children, aged 7–18, and their parent(s) participated: 40 children had a primary DSM-IV-TR classification of ASD, and 40 children had a primary DSM-IV-TR classification of ADHD (based on a multi-disciplinary consensus diagnosis, see procedure). No standardized measures were used to confirm the primary diagnosis. However, we are confident about the reliability of the ASD classification since in another sample of children with ASD, partly from the same outpatient mental health care center, the agreement between a clinical DSM-IV-TR classification of ASD and an ASD classification based on the Autism Diagnostic Interview-Revised (ADI-R; Lord et al. 1994) was excellent (97.8 %; van Steensel, Bögels and de Bruin, submitted). In addition, the KID-SCID (Hien et al. 1994; Dutch translation by Dreessen et al. 1998) was used to assess psychiatric comorbid disorders of which one is ADHD. For 36 out of the 40 children (90 %) classified as having ADHD, an ADHD- diagnosis was confirmed by the KID-SCID. That means that four children did not meet KID-SCID criteria for ADHD. It was decided not to exclude these children because—after disclosure of the KID-SCID results—these children still received a DSM-IV based diagnosis of ADHD as established by the multi-disciplinary team based on other diagnostic criteria (e.g. the cognitive profile on the Wechsler Intelligence Scale for Children-Revised, WISC-R (Wechsler 1974; van Haasen et al. 1986), the ratings on the Child Behavior Check List, CBCL (Achenbach 1991), and/or the impairment of attention problems for those children in daily functioning).

Of the children with ASD, 28 (70.0 %) were diagnosed with PDD-NOS and 12 children were diagnosed with Aspergers’ Syndrome (30.0 %). Of the ADHD sample, 23 children were classified as having ADHD-combined type (57.5 %), nine as having ADHD-inattentive type (22.5 %) and five as having ADHD-hyperactivity type (12.5 %). For three children (7.5 %), DSM-IV-TR classification of ADHD was postponed (i.e. a formal diagnosis of ADHD was not received because families dropped out)**.** However, we decided not to exclude those three children from the study because they all met DSM-IV criteria of ADHD based on the KID-SCID. None of the children in the ADHD sample were suspected of having ASD.

The ASD sample consisted of 36 boys and four girls with a mean age of 11.10 years (range = 8–18 years; SD = 2.82). The ADHD sample consisted of 37 boys and three girls and had a mean age of 11.13 years (range = 7–17 years; SD = 2.85). No group differences were found for gender, *X* ^2^ (1) = 0.157; *p* = .692, or age, *F* (1, 78) = 0.002; *p* = .969. For a summary of the education level and cognitive functioning of both groups, see Table 2. No differences between groups were found for primary education level, *X* ^2^(1) = 0.005; *p* = 1.00, secondary education level, Mann-Whitney U = 43.50; *p* = .260, or level of cognitive functioning, Mann–Whitney U = 487.50; *p* = .371.

**Table 2:** Education level and cognitive functioning of the ASD and ADHD sample

	ASD (*n* = 40)		ADHD (*n* = 40)	
	*n*	%	*n*	%
School^a^				
*Primary (elementary)*				
Special	2	5.0	2	5.0
Regular	28	70.0	26	65.0
*Secondary*				
Special	1	2.5	4	10.0
Low level	1	2.5	1	2.5
Moderate level	3	7.5	3	7.5
High level	3	7.5	3	7.5
*Vocational*				
Low level	1	2.5	1	2.5
Moderate level	0	0.0	0	0.0
High level	1	2.5	0	0.0
Cognitive functioning (IQ)				
50–69	2	5.0	0	0.0
70–79	4	10.0	3	7.5
80–89	0	0.0	5	12.5
90–110	16	40.0	11	27.5
111–120	9	22.5	11	27.5
121–130	3	7.5	0	0.0
>130	3	7.5	0	0.0
IQ data not available	3	7.5	10	25.0

^a^Schools in the Netherlands are divided in primary, secondary and vocational schools. Secondary and vocational schools are split into three education levels: low, moderate and high. Special education is viewed as a separate category; however, all levels (low-moderate-high) may be present. After graduation, transfers are possible (1) from a lower to a higher level within secondary or vocational schools and (2) from a secondary to a vocational school with the same level

### Instrument

The KID-SCID (Hien et al. 1994; Dutch translation by Dreessen et al. 1998) was used to assess (comorbid) psychiatric DSM-IV disorders. The KID-SCID is based on the widely used adult SCID with the questions adapted for applicability to children (and with additional childhood disorders). The adult SCID has demonstrated acceptable reliability and validity levels (e.g. Basco et al. 2000; Lobbestael et al. 2011; Spitzer et al. 1992; Williams et al. 1992). In addition, several studies support the validity and reliability of the KID-SCID (Matzner 1994; Matzner et al. 1997; Smith et al. 2005; Trimbremont et al. 2004). Kappa values for the test–retest reliability of the KID-SCID were found to range from .63 to .84 for the disruptive behavior disorders (kappa was .84, .63, and .84 for ADHD, ODD, and CD respectively) and from .44 to 1.0 for the anxiety disorders (kappa was 1.0, .66, and .44 for social anxiety disorder, separation anxiety disorder, and post-traumatic stress disorder, respectively) (Matzner et al. 1997). In addition, kappa values for the inter-rater reliability were 1.0 for disruptive behavior disorders, .63 for anxiety disorders, and .76 for the diagnosis of a current major depressive disorder (Trimbremont et al. 2004).

In administering the KID-SCID, a respondent is asked whether a particular DSM-IV symptom is present. The symptom is rated as: (1) absent, (2) possibly present, or (3) present. Both child and parent(s) provide answers and the interviewer combines the information of all respondents to rate a ‘best’ score. Next, the number of symptoms rated as ‘present’ is counted. If the required number of symptoms is met (i.e. the DSM-IV symptom-criterion is met), the interviewer asks about other DSM-criteria (such as ‘at what age symptoms emerged’ or ‘whether symptoms cause interference with daily activities’). Finally, a KID-SCID diagnosis is obtained when all DSM-IV criteria are met. In the present study, information was obtained from parent(s) and child and the following sections of the KID-SCID were administered: disruptive behavior disorders (ADHD, ODD and CD), mood disorders (major depressive disorder and dysthymic disorder) and anxiety disorders (separation anxiety disorder, social anxiety disorder, specific phobia, generalized anxiety disorder, obsessive–compulsive disorder, panic disorder, agoraphobia, post-traumatic stress disorder, and anxiety disorder not otherwise specified).

### Procedure

DSM-IV classification of ASD or ADHD was established by a multi-disciplinary team of psychologists, therapists, social workers and child psychiatrists, and was based on clinical evaluations. Clinical evaluations included for example interviews with parent(s) and child, observations of child-parent interaction, school observations, diagnostic assessments (e.g. WISC-R, Wechsler 1974; Van Haasen et al. 1986; CBCL, Achenbach 1991) and/or psychiatric consults. Approval was given by the medical-ethical committee, and participants signed informed consent. The KID-SCID was part of the intake procedure in the outpatient mental health center and was administered by psychologists who had multiple years of experience with the assessment and treatment of children with psychiatric disorders. In addition, the interviewers were trained by the translators of the KID-SCID who themselves were extensively trained in the use of the adult SCID.

### Analyses

Frequency counts of the psychiatric comorbid disorders for both groups were calculated. First, the overall comorbidity rate as well as the rates for internalizing and externalizing comorbid disorders in children with ASD and ADHD were compared using Chi-square analyses. If this test reached significance, then additional Chi-square analyses were conducted to explore group differences within the internalizing (mood/anxiety disorders) or externalizing (ODD/CD) disorders. In addition, the relation between comorbidity and age and between comorbidity and IQ was explored with Spearman’s rho. Considering the explorative nature of the research questions, alpha levels were set at .05 for all analyses.

## Results

Children with ASD were classified as having a comorbid disorder if they met KID-SCID criteria for at least one comorbid disorder. In line with the CBCL manual (Achenbach 1991) we defined participants as having a comorbid externalizing disorder if they met KID-SCID criteria for ODD and/or CD. Participants were classified to have an internalizing disorder if they met KID-SCID criteria for at least one mood disorder (major depressive disorder or dysthymic disorder) and/or one anxiety disorder (separation anxiety disorder, social anxiety disorder, specific phobia, generalized anxiety disorder, obsessive–compulsive disorder, panic disorder, agoraphobia, post-traumatic stress disorder, or anxiety disorder not otherwise specified).

In Table 3 the frequencies of the comorbid disorders for both samples are displayed. In the ASD sample, twenty-three children (57.5 %) were classified as having a comorbid disorder; internalizing disorders (35.0 %) were somewhat more common than externalizing disorders (22.5 %). Sixteen children of the ADHD-sample (40.0 %) met criteria for a comorbid disorder (note that for children with ADHD a KID-SCID diagnosis of ADHD was excluded from this comorbidity rate); externalizing disorders (27.5 %) being somewhat more common than internalizing disorders (12.5 %).

**Table 3:** Frequency of comorbid psychiatric disorders in children with ASD and children with ADHD

	ASD (*n* = 40)		ADHD (*n* = 40)	
	*n*	%	*n*	%
Comorbid disorder^a^	23	57.5	16	40.0
Internalizing disorders^b^	14	35.0	5	12.5
*Anxiety disorders*	11	27.5	4	10.0
Separation anxiety disorder	1	2.5	2	5.0
Social anxiety disorder	4	10.0	1	2.5
Specific phobia	5	12.5	1	2.5
Generalized anxiety disorder	2	5.0	0	0.0
Obsessive–compulsive disorder	3	7.5	0	0.0
Panic disorder	1	2.5	0	0.0
Agoraphobia	0	0.0	0	0.0
Post-traumatic stress disorder	0	0.0	0	0.0
Anxiety disorder not otherwise specified	0	0.0	0	0.0
*Mood disorders*	5	12.5	2	5.0
Major depressive disorder	1	2.5	2	5.0
Dysthymic disorder	4	10.0	0	0.0
Externalizing disorders^c^	9	22.5	11	27.5
Oppositional defiant disorder	9	22.5	8	20.0
Conduct disorder	1	2.5	5	12.5
Attention deficit hyperactivity disorder	9	22.5	36	90.0^d^

^a^Participants met KID-SCID criteria for at least one comorbid disorder (for the ADHD sample a KID-SCID diagnosis of ADHD was excluded)

^b^Participants met KID-SCID criteria for at least one anxiety disorder and/or mood disorder

^c^Participants met KID-SCID criteria for ODD and/or CD

^d^Group assignment was based on clinical diagnosis and therefore it was possible that not all participants with ADHD were having an ADHD diagnosis according to the KID-SCID

The two groups did not differ with respect to their overall rate of comorbid disorders, *X* ^2^(1) = 2.45; *p* = .117. Group differences were found to be significant for internalizing disorders, *X* ^2^(1) = 5.59; *p* = .018, but not for externalizing disorders, *X* ^2^(1) = 0.27; *p* = .606. Children with ASD had significantly more internalizing disorders compared to children with ADHD. Of the internalizing disorders, children with ASD were found to have significantly more anxiety disorders, *X* ^2^(1) = 4.02; *p* = .045, but not mood disorders, *X* ^2^(1) = 1.41; *p* = .432, compared to children with ADHD.

Correlations between comorbidity and age, and between comorbidity and IQ, are displayed in Table 4. Correlations were only small and none were found to be significant (all *p*’s > .10) indicating no relation between the presence of comorbidity and age, or between the presence of comorbid disorders and IQ.

**Table 4:** Correlations between comorbidity and age, and between comorbidity and IQ

	Total (*n* = 80)			ASD (*n* = 40)			ADHD (*n* = 40)		
	COMORBID	INT	EXT	COMORBID	INT	EXT	COMORBID	INT	EXT
Age	.07	.03	.10	.17	.23	.06	.00	−.21	.15
IQ^a^	−.01	.15	−.11	.07	.19	−.05	−.19	−.05	−.18

*COMORBID* having at least one comorbid disorder (for the ADHD sample a diagnosis of ADHD was excluded), *EXT* having at least one externalizing disorder (ODD/CD), *INT* having at least one internalizing disorder (anxiety/mood disorder)

^a^IQ-data was not available for three children with ASD and for ten children with ADHD

## Discussion

The present study examined psychiatric comorbidity in children with ASD and compared this to a group of children with ADHD. This was the first study to compare these two groups with respect to the frequencies of comorbid disorders (instead of symptoms) with a structured diagnostic interview. Children with ASD did not differ from children with ADHD with respect to their overall comorbidity rate. However, anxiety disorders were more often present in children with ASD compared to children with ADHD.

The finding that children with ASD and ADHD do not differ with respect to their overall comorbidity rate is not very surprising, since a review of comorbidity in children with ADHD (Gillberg et al. 2004) reported similar comorbidity rates (60–100 %) to those found by studies focusing on children with ASD (63.3–96.4 %, see Table 1). However, the finding is interesting for the following two reasons. First, comorbidity rates of children with ASD have not been compared directly to children with ADHD before. In addition, comparing the comorbidity rates between studies that have included children with ASD versus children with ADHD is difficult because of the wide variability in study designs, measurements to assess psychiatric comorbidity and study samples (i.e. community-based versus clinic-based samples). Secondly, the present findings suggest that children with ASD and children with ADHD are equally likely to develop comorbid psychiatric disorders, although specific comorbid disorders may be more present in one group than the other.

In the present study it was found that children with ASD exhibit more anxiety disorders compared to children with ADHD. This may partly be due to overlap between the core symptoms of ASD and symptoms of anxiety, particular in the case of obsessive–compulsive disorder and social anxiety disorder [see for example Wood and Gadow (2010) and van Steensel et al. (2011) for some discussion about this issue]. Nevertheless, our finding that children with ASD would have more anxiety disorders is in agreement with the study of Gadow et al. (2009) which found children with ASD to have more anxiety symptoms compared to children with ADHD. In addition, anxiety symptoms are usually not the core deficits in children with ASD, however, they respond favorably to treatment and extensive protocols for pharmacotherapy, and behavioral therapy for these additional anxiety symptoms in ASD is available (e.g. Posey and McDougle 2000; Santosh and Baird 2001). It is thus important to be aware of these comorbid symptoms, because not only are they treatable (whereas the core ASD symptoms are not), *not* treating them may lead to extra impairments in daily life skills such as completing school work or engaging in social situations. In addition, it is commonly found that children with ASD have higher levels of anxiety compared to typically developing children (e.g. Kim et al. 2000), however, for children with ADHD, as compared to typically developing children, these findings are more mixed (e.g. Biederman et al. 1991; Gau et al. 2010; van den Heuvel et al. 2007).

Also in line with the study of Gadow et al. (2009), we found that children with ASD and ADHD do not differ with respect to comorbid externalizing disorders (ODD/CD). In addition, in general, comorbidity between ADHD and ODD/CD is higher than between ADHD and internalizing disorders (e.g. Gillberg et al. 2004). Of note, in post hoc analyses we also found that children with ASD and a comorbid diagnosis of ADHD (*n* = 9) were significantly more likely to have ODD/CD (but not internalizing disorders) compared to those children with ASD without a comorbid ADHD-diagnosis (*n* = 31).

In contrast, we did not find higher levels of mood disorders in children with ASD. Note also that in Gadow et al. (2009) symptoms instead of disorders were assessed, which may explain the disagreement. Important to add here is that our study sample consisted of school-aged children only. From studies of Ghaziuddin et al. (2002) it is known that the rate of depression in ASD may rise with age. From the adolescent phase, and even more so for (young) adults, depression (i.e. a mood disorder) is the most common comorbid disorder in people with ASD (Ghaziuddin et al. 2002). It is therefore likely that our findings regarding comorbidity are not representative for the different stages of a life with ASD. Possibly, differences between ADHD and ASD become more prominent later in life (Roeyers et al. 1998).

For children with ASD specifically, it was found that 57.5 % suffered from comorbid disorders. This percentage is somewhat lower compared to the rates found in comparable studies (63.3–96.4 %, see Table 1). A first explanation for the relatively low rate we found is that we included only internalizing and externalizing disorders, excluding disorders such as enuresis, encopresis, chronic tic disorder, Tourette’s syndrome, and schizophrenia, which were included in several previous studies. A second explanation for the relatively low percentage of comorbidity might be related to the center to which the children of our sample were referred to. This was a general outpatient mental health clinic, not particularly specialized in ASD. As such, children with a large heterogeneity in psychiatric problems were referred, and possibly the more severe or complex cases (with higher comorbidity) were referred to more specialized centers. A third explanation for the relatively low comorbidity rate is that in most other studies different standardized interviews were used (e.g. the Diagnostic Interview Schedule for Children [DISC-IV; Ferdinand and Van der Ende 1998; Shaffer et al. 1996] in the study of De Bruin et al. 2007). The KID-SCID, similar to the DSM-IV, includes a criterion that the symptoms of ADHD, separation anxiety disorder, social anxiety disorder and generalized anxiety disorder may not be explained by the presence of an ASD. This criterion may have lowered the comorbidity rates of those disorders in the current study. Nonetheless, the percentage of comorbidity in children with ASD found in the current study (57.5 %) is still much higher compared to the rates (9.5–14.5 %) commonly found in typically developing children (e.g. Meltzer 2007; Ravens-Sieberer et al. 2008; Scholte et al. 2008).

Finally, limitations of the study need to be addressed. First, a larger sample size would have more power to detect possible influences of age and IQ. For example, the sample size of the current study was too small to examine possible influences of age or IQ for each specific disorder individually (e.g. social anxiety disorder, dysthymic disorder, etc.). In addition, while we did not find a significant relation between age (or IQ) and comorbidity in the present study, other studies did find differences across specific disorders for the influence of age in children with ASD (e.g. Matilla et al. 2010 found that behavioral disorders in children with ASD decreased with age, and Gjevik et al. 2011 found children with ASD and comorbid obsessive–compulsive disorder to be significantly older compared to those without obsessive–compulsive disorder). Further, the influence of other factors such as gender or ASD/ADHD subtype would be interesting to examine, however, this was not possible in the present study because of power issues. A second limitation is the overlap between ASD and ADHD. That is, a number of children with ASD (*n* = 9, 22.5 %) also had ADHD (according to KID-SCID criteria) and were found to differ from children with ASD without ADHD with respect to the rates of comorbid externalizing disorders. None of the children with ADHD were suspected to have ASD, however, although we have not assessed ASD symptoms in children with ADHD in the present study, it is likely that some of these children had (sub-) clinical levels of ASD symptoms. That is, other studies (e.g. Kochhar et al. 2011) have found higher levels of ASD traits in children with ADHD compared to typically developing children, and a substantial proportion of those children with ADHD reached screening thresholds predictive for an ASD diagnosis. It is possible that such overlap blurs the comparison between the two groups. Third, with respect to the diagnostic instrument (KID-SCID) it needs to be noted that psychometric studies are still ongoing. Results of studies examining the KID-SCID so far yielded fair to excellent test–retest reliability and inter-rater reliability (Matzner 1994; Matzner et al. 1997; Smith et al. 2005; Trimbremont et al. 2004), however, studies examining the validity of the KID-SCID are rare. A last limitation of the study is that we did not confirm ASD classification by standardized assessments such as the ADI-R (Lord et al. 1994) or the Autism Diagnostic Observation Schedule Generic (ADOS-G; Lord et al. 1999).

To the authors’ knowledge, this was the first study to examine the rate of psychiatric disorders in children with ASD and compared this to a group of children with ADHD, by using a structured and standardized diagnostic interview. It was found that children with ASD were equally likely to suffer from mood disorders and externalizing disorders (ODD/CD) as children with ADHD; however, they showed significantly more anxiety disorders compared to children with ADHD. Considering the large comorbidity found in children with ASD (and ADHD), clinicians should be alert of, and ideally, should always screen for comorbid disorders. Not only may these disorders interfere with daily functioning over and above the core symptoms of ASD, they may also exacerbate ASD-symptoms (Wood and Gadow 2010). However, reliable and valid screening instruments for assessing comorbidity as well as effective treatment programs for treating comorbidity in the ASD population are yet to be established. Note that there is a small but growing body of evidence to suggest that cognitive-behavioral therapies are effective for the treatment of anxiety disorders in youth with high-functioning ASD (e.g. Chalfant et al. 2007; Sofronoff et al. 2005; Wood et al. 2009), however, additional research is needed to examine the long-term follow up and factors that might influence treatment outcome. Furthermore, research concerning prevention programs as well as treatment options for the more challenging cases (e.g. children with intellectual disabilities or multiple comorbid disorders) is specifically needed.
